# Genetic testing of Behçet’s disease using next-generation sequencing to identify monogenic mimics and HLA-B*51

**DOI:** 10.1093/rheumatology/kead628

**Published:** 2023-11-25

**Authors:** Alice Burleigh, Ebun Omoyinmi, Charalampia Papadopoulou, Eslam Al-Abadi, Ying Hong, Fiona Price-Kuehne, Elena Moraitis, Hannah Titheradge, Francesca Montesi, Diane Xu, Despina Eleftheriou, Paul Brogan

**Affiliations:** Infection, Immunity and Inflammation, University College London Great Ormond Street Institute of Child Health, London, UK; Centre for Adolescent Rheumatology Versus Arthritis at University College London, London, UK; Infection, Immunity and Inflammation, University College London Great Ormond Street Institute of Child Health, London, UK; Paediatric Rheumatology, Great Ormond Street Hospital for Children NHS Foundation Trust, London, UK; Childhood Arthritis and Rheumatic Diseases Unit, Birmingham Women's and Children's Hospital NHS Foundation Trust, Birmingham, UK; Infection, Immunity and Inflammation, University College London Great Ormond Street Institute of Child Health, London, UK; Infection, Immunity and Inflammation, University College London Great Ormond Street Institute of Child Health, London, UK; Paediatric Rheumatology, Great Ormond Street Hospital for Children NHS Foundation Trust, London, UK; Clinical Genetics, Birmingham Women's and Children's Hospital NHS Foundation Trust, Birmingham, UK; Clinical Sciences Department, University of Birmingham, Birmingham, UK; Infection, Immunity and Inflammation, University College London Great Ormond Street Institute of Child Health, London, UK; Infection, Immunity and Inflammation, University College London Great Ormond Street Institute of Child Health, London, UK; Infection, Immunity and Inflammation, University College London Great Ormond Street Institute of Child Health, London, UK; Centre for Adolescent Rheumatology Versus Arthritis at University College London, London, UK; Paediatric Rheumatology, Great Ormond Street Hospital for Children NHS Foundation Trust, London, UK; Infection, Immunity and Inflammation, University College London Great Ormond Street Institute of Child Health, London, UK; Paediatric Rheumatology, Great Ormond Street Hospital for Children NHS Foundation Trust, London, UK

**Keywords:** Behçet’s disease, autoinflammatory disease, HLA typing, Exomiser, whole-exome sequencing

## Abstract

**Objective:**

Several monogenic autoinflammatory disorders and primary immunodeficiencies can present early in life with features that may be mistaken for Behçet’s disease (BD). We aimed to develop a genetic analysis workflow to identify rare monogenic BD-like diseases and establish the contribution of HLA haplotype in a cohort of patients from the UK.

**Methods:**

Patients with clinically suspected BD were recruited from four BD specialist care centres in the UK. All participants underwent whole-exome sequencing (WES), and genetic analysis thereafter by (i) examining genes known to cause monogenic immunodeficiency, autoinflammation or vasculitis by virtual panel application; (ii) scrutiny of variants prioritized by Exomiser using Human Phenotype Ontology (HPO); (iii) identification of copy number variants using ExomeDepth; and (iv) HLA-typing using OptiType.

**Results:**

Thirty-one patients were recruited: median age 15 (4–52), and median disease onset age 5 (0–20). Nine/31 (29%) patients had monogenic disease mimicking BD: five cases of Haploinsufficiency of A20 with novel *TNFAIP3* variants (p.T76I, p. M112Tfs*8, p. S548Dfs*128, p. C657Vfs*14, p. E661Nfs*36); one case of ISG15 deficiency with a novel nonsense variant (*ISG15*: p.Q16X) and 1p36.33 microdeletion; one case of common variable immune deficiency (*TNFRSF13B*: p.A181E); and two cases of TNF receptor-associated periodic syndrome (*TNFRSF1A*: p.R92Q). Of the remaining 22 patients, eight (36%) were HLA-B*51 positive.

**Conclusion:**

We describe a novel genetic workflow for BD, which can efficiently detect known and potentially novel monogenic forms of BD, whilst additionally providing HLA-typing. Our results highlight the importance of genetic testing before BD diagnosis, as this has an impact on choice of therapy, prognosis and genetic counselling.

Rheumatology key messages

- A significant minority of the UK BD patient population has an underlying monogenic disease rather than true BD.
- Whole-exome sequencing can identify underlying monogenic diseases and determine the HLA type in patients with suspected BD.
- Next-generation genetic sequencing should be considered for all UK patients with suspected BD before conferring this lifelong diagnostic label.

## Introduction

Behçet’s disease (BD) is a rare multisystemic variable vessel vasculitis, with a chronic and relapsing disease course [1]. The widespread vascular autoinflammation in BD can cause a range of debilitating symptoms, including oral and genital ulcers, uveitis, skin rashes and arthritis as cardinal features, while almost any organ can be affected, including the gastrointestinal, cardiovascular, renal, pulmonary, musculoskeletal, and central nervous systems. The diagnosis of BD remains challenging as there is marked heterogenicity in the clinical presentation.

The exact aetiology of BD is unknown. Several studies have demonstrated a multifactorial polygenic contribution to BD. Genome-wide association studies (GWAS) in cohorts around the world have defined multiple risk alleles across various loci which predispose individuals to the disease [2]. The most strongly associated genotype is Human Leucocyte Antigen (HLA)-B*51, a finding which has been replicated in numerous studies [3]. Despite this, HLA-B*51 genotype frequency varies significantly between cohorts and there remains a significant proportion of patients with BD where no risk allele positivity is identified. In addition, a significant limitation of GWAS is that it focuses on common variants, while rare genetic variants with allele frequencies <1% are excluded from association tests. Thus, whilst GWAS can provide important insights into how genetic variation impacts complex traits and diseases, these studies applied to patients with BD would not detect any of the rare monogenic autoinflammatory disorders and primary immunodeficiencies that can also present early in life with features that can be mistaken for BD. Distinguishing polygenic forms of BD from an ever-expanding number of monogenetic disorders masquerading as BD [4–17] is clinically important as their treatment can differ significantly [18]. Additionally, the determination of a potential monogenetic cause, whether treatable or not, has significance from the point of view of prognostication, genetic counselling, and in some instances avoidance of unnecessary and potentially harmful immunosuppression.

In this study, we hypothesized that it would be possible to use a bespoke genetic workflow to classify patients with BD into three broad categories: (i) monogenic BD or BD mimic; (ii) HLA-B*51-positive BD; or (iii) HLA-B*51-negative BD. To explore this hypothesis, we used whole-exome sequencing (WES) to identify rare monogenic BD-like diseases and establish the contribution of polygenic variation (HLA haplotype) of BD in a cohort of paediatric and adult patients recruited from four specialist centres in the UK.

## Patients and methods

### Patient recruitment

Full ethical approval for this study was given by the National Research Ethics Service, Bloomsbury Committee (ethics number 08/H0713/82). Informed written consent and/or assent was obtained from all patients and/or parents included in the study. A total of 31 patients with a working clinical diagnosis of BD (based on the expert local consultant physician or paediatrician diagnosis) were recruited from the following four hospitals in the UK providing highly specialist care for adults and children with BD: Great Ormond Street Hospital for Children (*n* = 17); Birmingham Women’s and Children’s Hospital (*n* = 10); the National Amyloidosis Centre at The Royal Free Hospital, London (*n* = 3); and The Royal London Hospital (*n* = 1). Patients with a clinical diagnosis of BD were recruited between November 2019 and November 2021 on a first-come, first-served basis. Clinical and laboratory data were collected using a data extraction form with pre-specified fields (corresponding to the data in Table 1), completed by the referring clinician and anonymized before return to us. Three BD definition scores were collated to explore their performance and relation to ultimate genotype in this series: the International Criteria for BD [20], the International Study Group criteria [21], and the Paediatric Behçet’s Disease (PEDBD) classification criteria [22]. A full description of all three sets of criteria is included in Supplementary Table S1, available at *Rheumatology* online.

**Table 1.** Clinical features, genetic findings, and clinical outcomes for 31 patients in the cohort

Case	Age^b^	Sex	Ethnicity	Clinical features^c^	Gene	Nucleotide change^d^	Amino acid change^d^	Predicted pathogenicity^e^	Zygosity	Variant classification	Identification method^f^	HLA-B*51	Genetic and clinical outcome^g^
1	17	M	Turkish	Oral ulcers, genital ulcers, necrotising folliculitis, acneiform lesions, arthritis, recurrent fevers, conjunctivitis. Similar features in mother.	—	—	—	—	—	—	—	No	***Polygenic BD.*** Responded well to short course of prednisolone.
2^a^	7	F	Asian	Oral ulcers, genital ulcers, recurrent fevers, anterior uveitis. Similar features in sister aged 3 years old.	—	—	—	—	—	—	—	No	***Polygenic BD.*** Good response to short course of prednisolone.
3^a^	15	F	White British	Oral ulcers, one episode of genital ulcers, recurrent fevers, headaches, fatigue, uveitis, retinitis, enlarged cervical glands. Mother had oral ulcers as a child.	—	—	—	—	—	—	—	Yes	***Polygenic BD (HLA-B*** *51+).Partial response to azathioprine but worsening of ocular inflammation prompted escalation to adalimumab with good effect.
4^a^	24	F	Pakistani	Oral ulcers, protracted diarrhoea, pulmonary embolism, erythema nodosum, polyarthritis, recurrent fevers, genital ulcers on one occasion, dry eyes, skin nodules, ulcerating lesions in nostrils. Similar features in two sisters and maternal aunt and uncle, fatal in all four cases.	—	—	—	—	—	—	—	No	***Polygenic BD.*** Poor response to colchicine; short course of methotrexate and tocilizumab stopped due to side effects, partial response to prednisolone. Anti-TNFα suggested but patient refused treatment.
5^a^	31	M	White Bulgarian	Oral ulcers, arthralgia, redness of the eyes, arthritis, erythematous maculopapular rash.	*TNFAIP3*	227C>T	p.T76I	D/B/D	Het	3	IP and Exomiser (0.967)	No	***Haploinsufficiency of A20.*** Partial response to colchicine, stopped due to intolerance and transaminitis. Now serial monitoring of inflammatory markers to decide about further management.
6^a^	9	F	Arab	Oral ulcers, recurrent fevers, headaches, abdominal pain, nausea, arthralgia, neutropenia, hepatosplenomegaly. Similar features in brother and cousin.	—	—	—	—	—	—	—	No	***Polygenic BD.*** Partial response to colchicine with improvement in fevers but persistent oral ulcers.
7^a^	15	F	White British	Oral ulcers, genital ulcers, anterior uveitis, recurrent episodes of fever, arthralgia, panniculitis rash, headaches, abdominal pain, nausea, and vomiting. Mother has Sjögren’s syndrome.	*TNFRSF13B*	542C>A	A181E	T/P/A	Het	5	IP	No	***Common variable immune deficiency.*** Good response to colchicine with resolution of ulcers, skin rashes and articular symptoms. Functional investigations to confirm relevance of *TNFRSF13B* variant are ongoing.
8^a^	14	M	White British	Recurrent episodes of oral ulcers, fevers, upper respiratory tract infections, urticarial skin rash, conjunctivitis, balanitis, headaches, and excessive vomiting. Focal mature ischaemic injury in the right cerebellar hemisphere. Brother has Crohn’s disease.	*TNFRSF1A*	362G>A	R121Q (R92Q)	T/B/N	Het	3 (LP)	IP, SP	No	***TRAPS.*** Good response to short course of prednisolone with fever and ulcer resolution.
9^a^	15	M	White British	Oral ulcers, uveitis, celullitic rash, episodes of fever, headache, and eye floaters.	—	—	—	—	—	—	—	Yes	***Polygenic BD (HLA-B*** *51+). Good response to colchicine with improvement of ulcers, skin rashes and no uveitis.
10	15	M	White British	Oral ulcers and genital ulcers. Similar features in mother.	*TNFAIP3*	946G>A	G316S	T/P/D	Het	3	IP and Exomiser (0.749)	No	***Haploinsufficiency of A20.*** Good response to short course of prednisolone followed by azathioprine maintenance with resolution of oral and genital ulcers.
						1625_1626insGAC TACAGCAGAGGCC	S548Dfs*128	-/-/-	Het	3	IP and Exomiser (0.998)		
11	17	M	White British	Oral ulcers, genital ulcers, abdominal pain, diarrhoea, arthralgia, hypermobile joints, necrotising folliculitis on trunk and lower limbs. Similar features in mother.	—	—	—	—	—	—	—	No	***Polygenic BD.*** No response to topical treatments or colchicine. Good response to azathioprine and the addition of infliximab. Persistent diarrhoea likely secondary to fructose intolerance.
12	18	M	White British	Oral ulcers, acneiform lesions, anterior and posterior uveitis, abnormal visual acuity, conjunctivitis, hypopyon, tinnitus, incidental brain microaneurysms.	—	—	—	—	—	—	—	Yes	***Polygenic BD (HLA-B*** * ***51+).*** Complete resolution of symptoms on azathioprine and infliximab.
13^a^	7	F	Italian/White British	Oral ulcers, genital ulcers, fever, and abdominal pain.	—	—	—	—	—	—	—	No	***Polygenic BD.*** Colchicine had no effect; a course of prednisolone resolved most symptoms.
14^a^	14	F	Pakistani	Oral ulcers, genital ulcers, headaches, chronic pulmonary interstitial emphysema, autoimmune hypothyroidism. Mother also has autoimmune hypothyroidism.	*TNFAIP3*	335_341del	M112Tfs*8	-/-/-	Het	3	IP and Exomiser (0.98)	No	***Haploinsufficiency of A20.*** Good response of genital ulcers to short course of prednisolone and azathioprine-now off treatment doing well, asymptomatic.
15^a^	4	F	White British	Genital ulcers, severe axilla and lower abdominal skin ulcers, lipodystrophy of the skin, recurrent fevers.	*ISG15*	46C>T	Q16X	././D	Het	3	IP and Exomiser (0.758)	No	***ISG15 deficiency.*** Topical steroid cream, antibiotics, antifungals, and methotrexate resolved symptoms.
					*SAMD11, NOC2L, KLHL17, PLEKHN1, PERM1, HES4, ISG15, AGRN, RNF223, C1orf159*	Deletion c.817341-1131023 (hg38)	—	-/-/-	Het	3	ExomeDepth, confirmed by TGMA		
16^a^	10	F	African	Oral ulcers, erythema nodosum, anterior uveitis, arthritis, recurrent fever, pulmonary hypertension, interstitial lung disease, cataracts. Sister (case 17) had similar symptoms.	—	—	—	—	—	—	—	No	***Polygenic BD.*** Persistent disease despite treatment with methotrexate, canakinumab and adalimumab. Better disease control with infliximab but treatment-resistant uveitis required off-license high dose.
17^a^	9	F	African	Oral ulcers, erythema nodosum, anterior uveitis, arthritis, recurrent fever, meningoencephalitis, raised intracranial pressure. Sister (case 16) had similar symptoms.	—	—	—	—	—	—	—	No	***Polygenic BD.*** Initial resolution of rash and fever on prednisolone and methotrexate but persistent joint disease and ocular inflammation prompted addition of adalimumab. Remained stable on adalimumab and methotrexate until development of adalimumab antibodies necessitated switch to infliximab and has remained well since.
18	12	M	Turkish	Oral ulcers, genital ulcers, arthralgia, recurrent fevers, conjunctivitis, headaches, abdominal pain. Similar features in mother and maternal grandfather.	—	—	—	—	—	—	—	Yes	***Polygenic BD (HLA-B*** *51+).Good response to colchicine treatment with resolution of all symptoms.
19	19	M	Romanian	Oral ulcers, genital ulcers, erythema nodosum, necrotising folliculitis, acneiform lesions, anterior uveitis, episodes of fever, arthralgia, headaches, dry cough, and chest pain.	—	—	—	—	—	—	—	Yes	***Polygenic BD (HLA-B*** *51+).Treated as sJIA initially with failure of methotrexate, anakinra and tocilizumab. Transient prednisolone dependant improvement. Adalimumab started on confirmation of BD diagnosis with almost immediate improvement and complete, steroid free resolution of symptoms.
20	10	M	White British	Oral ulcers, genital ulcers on one occasion, necrotising folliculitis, follicular lesions, episcleritis, occasional arthralgia. Father had BD with juvenile onset.	—	—	—	—	—	—	—	No	***Polygenic BD.*** One episode of mouth ulcers settled with fluticasone nasal spray. One episode of pseudofolliculitis that was mild and spontaneously resolved.
21^a^	12	M	White British	Oral ulcers, necrotising folliculitis, chronic anterior uveitis, pseudophakia, macular oedema, polyarthritis, generalized tonic clonic seizures, recurrent episodes of fever, tonsilitis, adenitis, recurrent diarrhoea, periods of poor weight gain. Younger brother (case 22) had similar symptoms. Mother has history of arthralgia and skin rashes.	—	—	—	—	—	—	—	No	***Polygenic BD.*** Good control on methotrexate and adalimumab combination therapy. Occasional mouth ulcers treated with fluticasone spray. Uveitis control improving and now only needs one drop Pred forte.
22^a^	10	M	White British	Oral ulcers, genital ulcers, recurrent fevers, arthralgia, urticarial rash, abdominal pain. Older brother (case 21) had similar symptoms. Mother has history of arthralgia and skin rashes.	—	—	—	—	—	—	—	Yes	***Polygenic BD (HLA-B*** *51+).Good response to colchicine initially but stopped due to intolerance. Infrequent mouth ulcers responsive to fluticasone spray. No recurrence of other symptoms for almost three years.
23^a^	17	M	Turkish	Oral ulcers, posterior uveitis, and severe, sight-threatening retinal vasculitis.	—	—	—	—	—	—	—	Yes	***Polygenic BD (HLA-B*** *51+).Initially treated with oral azathioprine and prednisolone. Worsening ocular inflammation prompted change to adalimumab to good effect.
24^a^	17	F	Turkish	Oral ulcers, genital ulcers, recurrent fevers, arthralgia, and fatigue.	—	—	—	—	—	—	—	No	***Polygenic BD.*** Good response to colchicine and topical corticosteroid treatment with resolution of all symptoms.
25^a^	50	M	White British	Oral ulcers, arthritis, headaches meningoencephalitis, deep vein thrombosis, proteinuria, IgA nephropathy with glomerulosclerosis. Father had history of recurrent oral ulcers. Eldest daughter has recently also developed oral ulcers.	—	—	—	—	—	—	—	No	***Polygenic BD.*** Previous trial of prednisolone, methotrexate, and hydroxychloroquine with only partial response. Good response to colchicine; no further symptoms and normalization of inflammatory markers.
26^a^	13	M	Pakistani	Oral ulcers, genital ulcers, recurrent fevers, erythema nodosum and arthritis. Father (case 27) had similar symptoms. Other five siblings asymptomatic.	—	—	—	—	—	—	—	No	***Polygenic BD.*** Persistent disease activity despite colchicine, prednisolone, and azathioprine. Achieved good control with adalimumab.
27^a^	52	M	Pakistani	Oral ulcers, genital ulcers, erythema nodosum, episode of sinus venous thrombosis and intracranial haemorrhage. Son (case 26) had similar symptoms. Other five children asymptomatic.	—	—	—	—	—	—	—	No	***Polygenic BD.*** Partial response to colchicine and short courses of oral prednisolone-persistence of genital and oral ulcers.
28^a^	18	M	Asian	Oral ulcers, genital ulcers, episcleritis, single episode of fever at presentation. Arthritis in both knees and one ankle, purpuric rash on feet, alopecia universalis, epididymo-orchitis, mild ventricular systolic dysfunction, oesophageal duplication cyst. Similar but milder features in sister.	—	—	—	—	—	—	—	Yes	***Polygenic BD (HLA-B*** *51+).Complete response to colchicine.
29	14	M	Welsh/Egyptian	Oral ulcers and perianal ulcers, episodes of fever, arthralgia, headaches, diarrhoea and vomiting, abdominal pain, tonsilitis, episode of acute renal failure (presumed kidney stone), enlarged vestibular aqueduct so wears cochlear implants. Mother had similar symptoms. Maternal grandmother, brother and maternal cousins had similar but milder symptoms.	*TNFAIP3*	1966dupC	C657Vfs*14	−/-/-	Het	3	IP and Exomiser (0.949)	No	***Haploinsufficiency of A20.*** Failed colchicine and azathioprine monotherapies. Initial fill improvement of adalimumab then azathioprine and infliximab combination, developed antibodies to anti-TNFα. Severe flare with fever, oral ulcers, perianal ulcers livedo reticularis and colitis requiring 8 weeks of tapering prednisolone and now stable and symptom free on ustekinumab off steroids. Mother, but not other mildly affected relatives, also found to have *TNFAIP3*: p.C657Vfs*14 mutation. This case is being further investigated for additional genetic cause of his aqueduct dilation.
30	41	F	White British	Oral ulcers, genital ulcers, one episode of anterior uveitis, papulopustular lesions on face and scalp, folliculitis, joint pain in knees, hips and lower back, recurrent headaches and migraines, diarrhoea, vomiting, occasionally blood in stool. Mother and sister had similar symptoms, including haemorrhagic stroke in the mother.	*TNFRSF1A*	362G>A	R121Q (R92Q)	T/B/N	Het	3 (LP)	IP, SP	No	***TRAPS.*** Symptoms worsened despite colchicine and azathioprine treatments, currently being treated with anti-TNFα with persistent symptoms. Because not all symptoms can be explained by a TRAPS diagnosis, this patient may have an additional genetic diagnosis.
31	6	M	White British	Perianal ulcers, recurrent fever, intermittent maculopapular rash, eczema, mild hepatosplenomegaly, abdominal pain and loose stools, lymphadenopathy, recurrent tonsilitis.	*TNFAIP3*	1979delG	E661Nfs*36	−/-/-	Het	3	IP and Exomiser (0.929)	No	***Haploinsufficiency of A20.*** Good control of symptoms with short courses of oral prednisolone.

a Family DNA was available and either Sanger sequencing or whole-exome sequencing was applied to reduce the variant shortlist through segregation analysis.

b Age at the time of this study.

c Relevant clinical features in other family members are detailed.

d Because each gene may have multiple splicing isoforms, the variants are annotated according to HUGO Gene Nomenclature Committee RefSeq transcript [19].

e Prediction (SIFT/Polyphen2/MutationTaster); B = benign, D = damaging or deleterious, P = probably damaging, T = tolerated, N = neutral, A = disease causing automatic for MutationTaster, . = termination, — = no prediction.

f For variants identified by Exomiser, Exomiser combined score is detailed in brackets. A higher score (0–1) indicates a more highly prioritized variant.

g Treatments administered at doses: oral prednisolone 1–2 mg/kg/day tapered over 6–8 weeks; oral colchicine 250 µg–1 mg twice daily; adalimumab 40 mg subcutaneously every 2 weeks; azathioprine 1–2 mg/kg/day oral; methotrexate 15 mg/m^2^ oral or subcutaneously weekly.

F: female; M: male; BD: Behçet’s Disease; Het: heterozygous; TRAPS: tumour necrosis factor receptor-associated periodic syndrome; LP: low penetrance; HLA: human leucocyte antigen; sJIA: systemic juvenile idiopathic arthritis; TGMA: targeted genomic microarray analysis; IP: inflammation panel; SP: somatic panel.

### Genetic analysis approach

**Figure 1.**
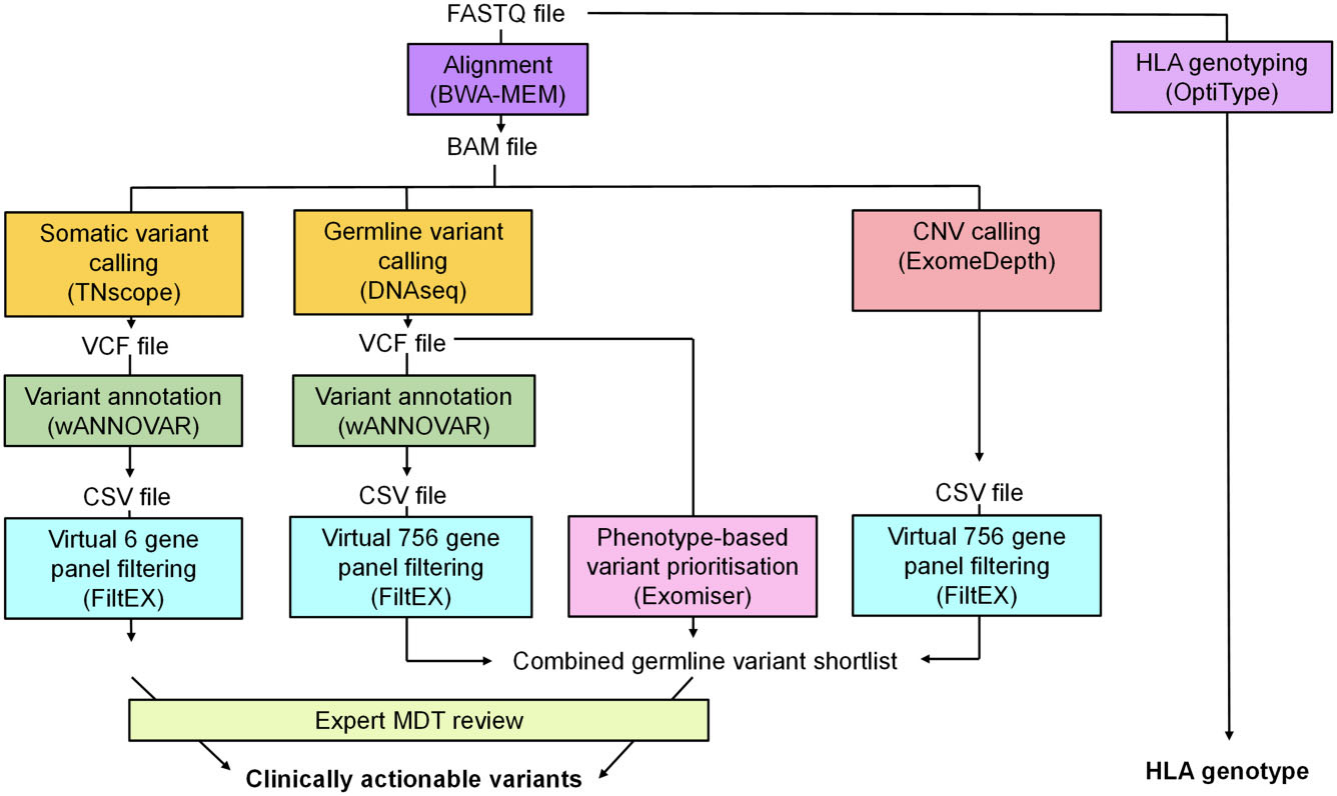
Bioinformatic genetic analysis summary. FASTQ files generated from whole-exome sequencing were aligned to human reference genome hg38 using BWA-MEM [23]. Variants were called from BAM files using TNscope [24, 25] for somatic variants; DNAseq for germline variants [24]; and ExomeDepth [26] for copy number variants (CNV). Somatic and germline VCF files were annotated using wANNOVAR [27]. CSV files were then filtered using virtual gene panels through an R script. The germline VCF file was also analysed using Exomiser [28]. The resulting variants were combined into a shortlist and reviewed by an expert multidisciplinary team (MDT) to identify clinically actionable variants. FASTQ files were also processed in OptiType [29] to determine HLA genotypes

Patient DNA was either obtained directly from the clinical genetic laboratories of the respective hospitals or extracted from whole blood using Gentra Puregene blood kit (Qiagen, Hilden, Germany), or from saliva using prepIT L2P kit (DNA Genotek Inc., Stittsville, Ontario, Canada). The genetic analysis workflow we designed is shown in Fig. 1. The aim of the workflow was to identify clinically actionable variants, including germline single nucleotide variants (SNVs), small indels and copy number variants (CNVs) and somatic SNVs and small indels in clinically relevant genes. To this end, three variant callers were applied, including Sentieon^®^ DNAseq^®^ [24] for germline SNVs and small indels, Sentieon^®^ TNscope^®^ [24, 25] for somatic SNVs and small indels, and ExomeDepth [26] for germline CNVs. These variants were then interpreted using either an in-house filtering workflow and virtual gene panel, or the application of Exomiser [28], a tool which prioritizes variants based on gene-phenotype associations [30] and predicted pathogenicity of variants. To obtain the HLA genotype, OptiType [29] was applied, a tool which accurately predicts HLA class I genotype from exome sequencing data.

### Whole-exome sequencing (WES) and bioinformatic analysis

DNA was quantified using a Qubit^®^ dsDNA High Sensitivity kit and a Qubit^®^ 2.0 fluorometer (Invitrogen, Waltham, Massachusetts, USA), and then shipped to Nonacus Ltd (Birmingham, UK) or sister company Informed Genomics Ltd (Birmingham, UK), for WES using their ExomeCG Cell3™ service (https://nonacus.com/exome-cg/). Data were processed by Nonacus Ltd or Informed Genomics Ltd using a best practice germline pipeline including unique molecular identifier (UMI) tag extraction, read alignment to GRCh38 using BWA-MEM [23], UMI consensus calling and re-alignment.

#### Germline variant calling

Genotyping was completed with Sentieon^®^ (San Jose, California, USA) DNAseq^®^ [24] across ExomeCG capture regions (extending ∼100 bp beyond exons). Variant quality annotation was applied using BCFtools [31]. Quality control was performed by Picard (http://broadinstitute.github.io/picard/) and coverage performance assessed by MosDepth [32].

#### Somatic variant calling

Genotyping was completed with Sentieon^®^ TNscope^®^ [24, 25] across ExomeCG capture regions (extending ∼100 bp beyond exons). Variant quality control was then performed as per germline variant calling.

#### Copy number variant calling

CNV calling was performed using ExomeDepth 1.1.10 [26] following best practice. Reference samples were chosen by best fit.

#### Variant annotation and shortlisting

In-house, the resulting VCF files were annotated using wANNOVAR (https://wannovar.wglab.org) which provided allele frequencies from public databases and in silico predictions of pathogenicity [27]. Variants were then filtered using an in-house R workflow, which is publicly available (https://github.com/AlBurl/FiltEX). This workflow applied multiple steps to filter variants: (i) synonymous variants were excluded; (ii) variants with a minor allele frequency >1% in population frequency databases 1000 Genomes (http://www.1000genomes.org), ExAC (http://exac.broadinstitute.org) and gnomAD (https://gnomad.broadinstitute.org) were excluded; and (iii) variants not present in the 756 genes of our in-house inflammation panel were removed, which combines our previously described panels [33, 34] (Supplementary Table S2, available at *Rheumatology* online) and is now routinely used for sequencing analysis in the NHS. For somatic variant filtering, a reduced 6-gene panel (*NLRC4*, *NLRP3*, *NOD2*, *STING1*, *TNFRSF1A*, *UBA1*) was applied, as somatic variants in these genes can be associated with diseases potentially mimicking BD [35–42]. CNVs were filtered by removing CNVs not containing at least one gene from the 756-gene inflammation panel (Supplementary Table S2, available at *Rheumatology* online). The germline VCF files were also analysed using Exomiser 12.1.0 [28], which was installed via GitHub (https://github.com/exomiser/Exomiser) and run using default settings. Relevant Human Phenotype Ontology (HPO) terms were selected on a case-by-case basis using the online database (https://hpo.jax.org/app/). The prioritized genes with an Exomiser score >0.75 were added to the shortlist of variants for each patient.

#### Expert multi-disciplinary team review of variant shortlists

The variant shortlist for each participant was manually reviewed by considering several parameters. Firstly, using the ClinVar database (https://www.ncbi.nlm.nih.gov/clinvar/), the variants were classified into pathogenicity groups (Class 1: benign; Class 2: likely benign; Class 3: unknown significance; Class 4: likely pathogenic; Class 5; pathogenic), according to the American College of Medical Genetics (ACMG) guidelines [43]. Next, the Online Mendelian Inheritance in Man (OMIM) database (https://omim.org/) was used to determine gene inheritance. Genes with autosomal recessive inheritance with only one heterozygous variant in the shortlist were also removed. From the remaining list, a multidisciplinary team (MDT) (A.B., E.O., P.B., D.E.) assessed the relevance of the genes to the reported phenotype. In cases where family DNA was available, the shortlist could be further reduced using family WES or Sanger sequencing data to assess the segregation of variants with the phenotype. Clinically actionable variants resulting in a molecular diagnosis were confirmed by Sanger sequencing and referred to the patient’s respective accredited genetic testing laboratory for validation. Primer sequences used for Sanger sequencing are available on request.

#### OptiType

OptiType [29], a tool for in-silico HLA typing, was installed via GitHub (https://github.com/FRED-2/OptiType) and run using DNA mode on unfiltered FASTQ files.

### Statistical testing

Fisher’s exact tests were performed using GraphPad Prism version 9 (GraphPad Software).

## Results

### Patient demographics and clinical manifestations

A total of 31 patients were recruited: 12 female individuals (39%) and 27 White British, European or Middle Eastern (87%), two Asian (6%) and two African (6%) individuals. The median age of the cohort was 15 years (range 4–52 years). The median age of disease onset, excluding the three cases without a defined age, was 5 years old (range 0–20 years), and 30 patients (97%) had disease onset under the age of 18 years. The clinical features observed in this cohort included: oral aphthosis (94%), recurrent fever (74%), genital aphthosis (67%), skin lesions (61%), arthritis/arthralgia (61%), ocular lesions (42%), headaches (39%) and other neurological features (16%). Twenty-eight patients (90%) fulfilled the ICBD criteria [20] and 14 patients (45%) fulfilled the International Study Group (ISG) criteria [21]. Of the 23 paediatric patients, 11 (48%) fulfilled the PEDBD criteria [22]. Full cohort demographics are detailed in Table 1, and a comparison of their BD diagnostic (ICBD, ISG) or classification (PEDBD) criteria scores in Supplementary Table S3, available at *Rheumatology* online.

### Genetic findings

WES and the genetic analysis workflow were performed for each of the 31 patients recruited to the study. After careful consideration of the variant shortlists by the expert MDT, and data obtained from WES or Sanger sequencing in other family members where this was possible, we identified nine patients that harboured variants considered to be causing a monogenic disease **(**Table 1**)**. None of these patients were HLA-B*51 positive.

**Figure 2.**
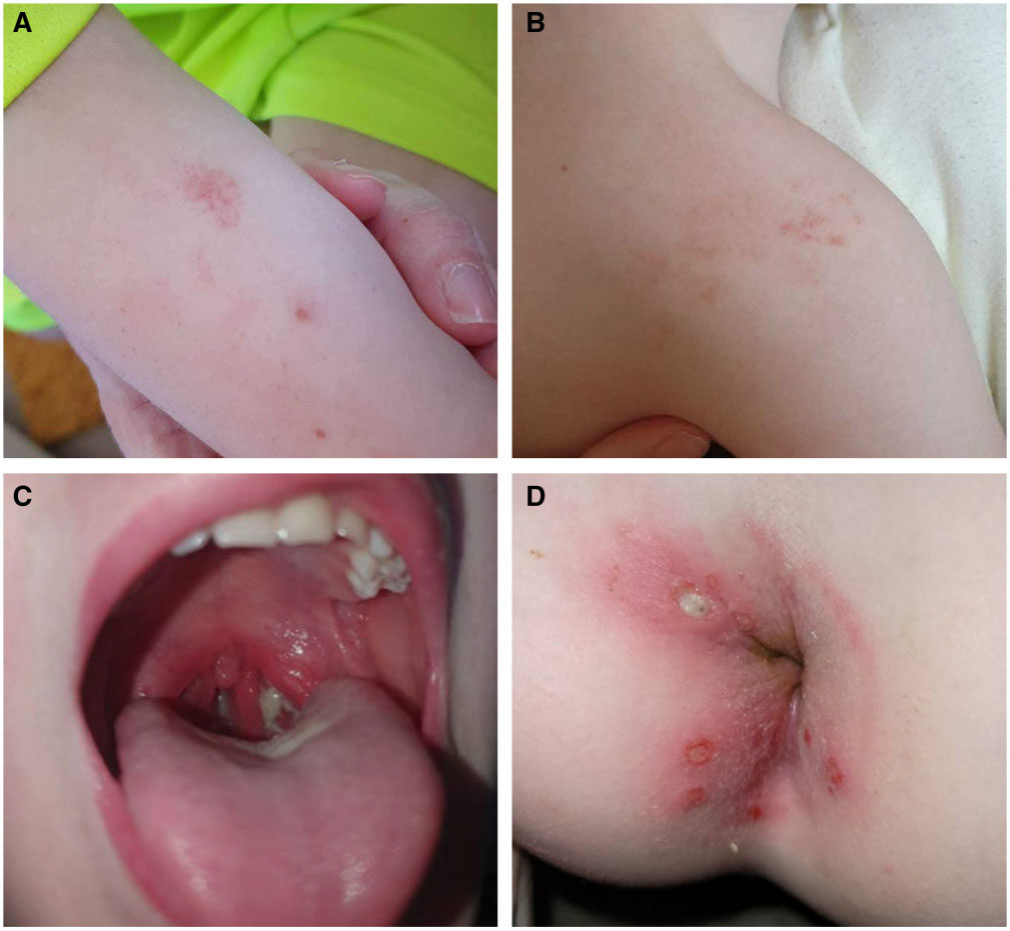
Clinical pictures of case 31. (**A**/**B**) Diffuse maculopapular rash with irregular borders and subtle telangiectasia affecting the forearm and shoulder, (**C**) bilateral exudative tonsillitis during a febrile episode, and (**D**) multiple discrete perianal lesions with abscess and fistula formation, and erythema and excoriation of the surrounding skin

Five patients (cases 5, 10, 14, 29, 31) were classified as having Haploinsufficiency of A20 (HA20), caused by five novel heterozygous variants in *TNFAIP3* (p. T76I, p. S548Dfs*128, p. M112Tfs*8, p. C657Vfs*14, p. E661Nfs*36). Cases 5, 10 and 14 presented mainly with recurrent episodes of oral and/or genital ulceration, case 5 also had an erythematous maculopapular rash and arthralgia and case 14 had autoimmune hypothyroidism and chronic interstitial pneumonia. Case 29 had severe disease characterized by oral and perianal ulcers, fever, arthralgia, colitis, an episode of acute renal failure and enlarged vestibular aqueduct requiring cochlear implants. He failed to respond to multiple treatments but ultimately responded to ustekinumab therapy that allowed him to wean off his prednisolone therapy and reduced flares. Case 31 presented in infancy with perianal ulceration first noticed at 2 weeks of age, followed by recurrent fevers from birth to 12 months of age associated with a maculopapular rash, lymphadenopathy, recurrent tonsilitis and raised inflammatory markers (Fig. 2). The pathogenicity of these variants was investigated for cases 5, 14, 29 and 31 using an NF-ĸB signalling assay [44] which showed upregulation of the pathway in all four cases (Supplementary Fig. S1, available at *Rheumatology* online). Case 10 was unavailable for follow-up testing.

Case 15 was diagnosed with ISG15 deficiency, caused by a heterozygous novel nonsense variant in *ISG15* (p.Q16X) on one allele, and heterozygous multigene 1p36.33 microdeletion on the other, resulting in complete loss of ISG15. She first presented at 5 months of age with ulceration of the groin, axilla, genitals and lower abdomen and progressive diffuse lipodystrophy. Of note, there were no concerns about her development, she had no seizures or abnormal neurology on clinical examination and neuroimaging of the brain revealed no intracranial calcification. These variants were confirmed by Sanger sequencing and targeted genomic microarray analysis, respectively.

Case 7 was diagnosed with common variable immune deficiency (CVID), as she was found to have a class 5 variant in *TNFRSF13B* (p.A181E). Immunoglobulin level testing confirmed low IgA [0.63 g/l, reference range (RR) 0.8–2.8 g/l] and IgM (0.47 g/l, RR 0.5–1.9 g/l), although her IgG levels were in the expected range (8.68 g/l, RR 5.4–16.1 g/l). The mother of this case, who had a diagnosis of Sjögren’s syndrome, was also found to possess this variant.

Two cases (8 and 30) had the same class 5 variant in *TNFRSF1A* (p.R121Q, also known as p.R92Q), associated with tumour necrosis factor receptor-associated periodic syndrome (TRAPS). Case 8 presented at 8 months of age with recurrent fevers and oral ulceration associated with headaches and vomiting. This prompted magnetic resonance imaging of the head, which showed a mature brain ischaemic injury which has remained unchanged on interval scanning. The patient subsequently developed recurrent episodes of fever associated with widespread urticarial skin rash, conjunctivitis and balanitis. Case 30 experienced recurrent oral ulcers in childhood, then later genital ulcers, papulopustular lesions, folliculitis, and alternating diarrhoea and vomiting. Because not all of her symptoms could be explained by a diagnosis of TRAPS, this patient may have an additional genetic diagnosis. The pedigrees and segregation of proposed disease-causing variants in the nine families classified as having monogenic disease are available in Supplementary Fig. S2, available at *Rheumatology* online.

Of the remaining cases, 8/22 (36%) were HLA-B*51 positive (cases 3, 9, 12, 18, 19, 22, 23, 28), higher than expected in the healthy population (allele frequency 3–8% in the UK [45]). The full HLA typing results of the cohort are detailed in Supplementary Table S4, available at *Rheumatology* online. The remaining 14 cases were classified as having early-onset likely polygenic BD, due to these patients lacking a discernible monogenic cause of disease, and the consistency of their presentations with a typical BD phenotype.

### Influence of ethnicity, age of disease onset and diagnostic criteria scores on genotyping results

**Figure 3.**
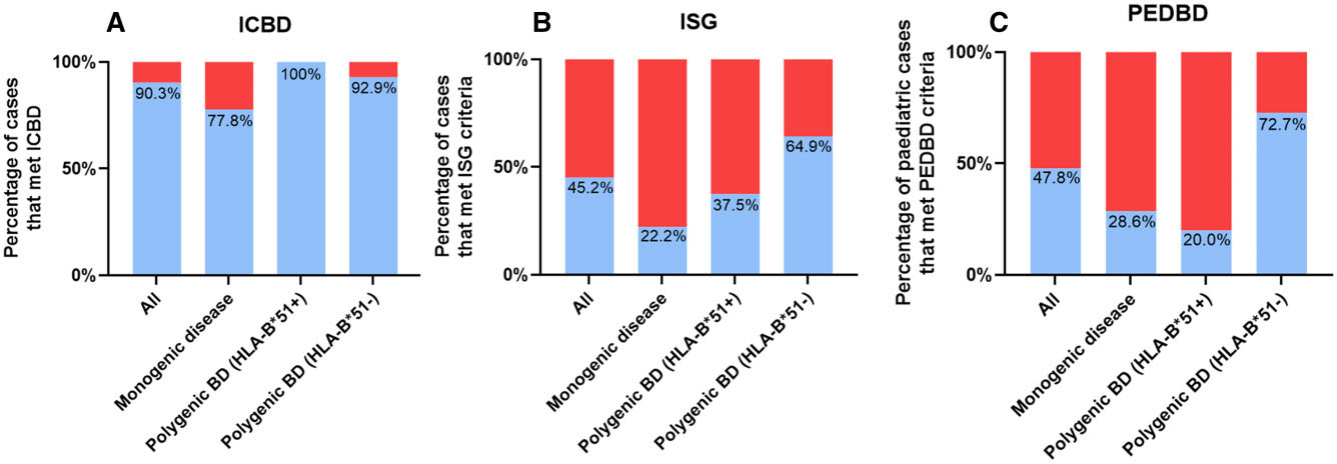
Comparison of the scores for the International Criteria for Behçet’s Disease (ICBD), the International Study Group (ISG) and the Paediatric Behçet’s Disease (PEDBD) criteria. Percentage of cases grouped by outcome of the genetic analysis that met the (i) International Criteria for Behçet’s Disease (ICBD) (score ≥4) [20], (ii) International Study Group (ISG) [21] criteria and (iii) Paediatric Behçet’s Disease (PEDBD) criteria (score ≥3) [22]. For ICBD and ISG criteria there were nine cases with monogenic disease; eight cases with HLA-B*51 associated Behçet’s Disease (BD): and 14 cases with likely polygenic BD. For PEDBD criteria, only paediatric cases were included, hence the analysis included 23 cases of which seven had monogenic disease, five cases had HLA-B*51 associated BD and 11 cases had likely polygenic BD

The proportions of each patient group, categorized by genetic outcome, that met the ICBD [20], ISG [21] and PEDBD [22] criteria are summarized in Fig. 3. Across all groups of patients, a higher proportion of patients met the ICBD criteria (77.8–100%) than the ISG criteria (22.2–64.9%) or PEDBD (20–72.7%). To investigate the influence of diagnostic criteria classification, ethnicity, and age of disease onset on genotyping results, Fisher’s exact tests were performed (Table 2). One of these tests was statistically significant, indicating that ethnicity (White British *vs* not White British) influenced genetic outcome in this cohort. The remaining tests were not statistically significant, suggesting that in this cohort, diagnostic criteria outcome and age of disease onset did not influence genetic outcome.

**Table 2.** Contingency tables comparing patients’ diagnostic criteria classification, ethnicity and age of disease onset with their genetic outcome

Test 1: Influence of ethnicity on genetic outcome P = 0.0155 (significant at P < 0.05)		
	Monogenic	Non-monogenic
White British	8	8
Not White British	1	14
**Test 2: Influence of age of disease onset on genetic outcome P = 0.290 (n.s.)**		
	Monogenic	Non-monogenic
Age of disease onset ≤16	8	22
Age of disease onset >16	1	0
Test 3: Influence of ethnicity on HLA-B*51 genotype P = 1 (n.s.)		
	HLA-B*51+	HLA-B*51-
White British	4	12
Not White British	4	11
**Test 4: Influence of ICBD classification on genetic outcome P = 0.195 (n.s.)**		
	Monogenic	Non-monogenic
ICBD met	7	21
ICBD not met	2	1
**Test 5: Influence of ISG classification on genetic outcome P = 0.132 (n.s.)**		
	Monogenic	Non-monogenic
ISG criteria met	2	12
ISG criteria not met	7	10
**Test 6: Influence of PEDBD classification on genetic outcome^a^ P = 0.371 (n.s.)**		
	Monogenic	Non-monogenic
PEDBD criteria met	2	9
PEDBD criteria not met	5	7

Fisher’s exact tests were performed with a significance level of 0.05.

a For PEDBD criteria comparisons, only paediatric cases were included.

n.s.: not significant.

## Discussion

In this study, we deployed a bespoke WES genetic workflow for the identification of monogenic mimics of BD and HLA genotyping to better describe the genetic architecture of BD in an unselected UK cohort. Nine/31 (29%) patients had monogenic disease presenting with a BD-like phenotype, and the majority of these (5/9, 56%) were HA20, the most well-described monogenic mimic of BD. None of the cases with monogenic diagnoses were HLA-B*51 positive; however, HLA-B*51 positivity was confirmed in 8/22 (36%) of the remaining non-monogenic cases, in keeping with typical and likely polygenic BD phenotype. Thus, application of this BD-specific genetic workflow stratified UK patients into three broad groups: monogenic BD mimics; likely typical polygenic BD with HLA-B*51 positivity; and likely typical polygenic BD without HLA-B*51 positivity. In this cohort, there was no significant influence of age of disease onset, or any of the three BD diagnostic criteria classification on genetic outcome, suggesting that these cannot be used as predictive factors for whether a patient harbours a monogenic mimic of BD. We observed an apparent influence of ethnicity (White British *vs* not White British) on genetic outcome, with White British patients more likely to have a monogenic outcome. Although this cohort was small, this may indicate that genetic testing of BD-like disease could be even more important in the UK than Eastern countries, perhaps because the prevalence of true polygenic BD is much lower here [46], increasing the chances of a monogenic cause of disease. Accordingly, these data strongly suggest that WES be performed in all UK patients with suspected BD before conferring this lifelong diagnostic label, since the genotype profoundly affects patient management.

We adopted a multi-pronged analysis approach involving scrutiny of a virtual germline panel of 756 genes known to cause immune deficiency, autoinflammation and vasculitis [33, 34]; Exomiser analysis using HPO terms to aid variant prioritization across the whole exome [28]; somatic variant gene panel analysis of six preselected genes (*NLRC4*, *NLRP3*, *NOD2*, *STING1*, *TNFRSF1A*, *UBA1*) known to be associated with disease states when present as somatic variants [35–42]; and read count CNV analysis [26]. For the nine patients with likely monogenic disease, the causative variant was identified by both the virtual application of the inflammation gene panel list and further Exomiser analysis in six cases; and in three cases the variant was identified only by targeted scrutiny of the gene panel (Table 1). The Exomiser suite comprises several methods that use clinical data, model organism phenotype data, as well as random-walk analysis of protein interactome data to perform genetic variant prioritization and facilitate automation in disease-gene discovery and diagnostics. We demonstrate that the combination of the application of the Exomiser suite analysis together with a bespoke virtual gene panel application creates a more robust ‘belt and braces’ approach for genetic diagnosis of rare diseases. Additionally, for the ISG15 deficient patient with compound heterozygous variants, the multigene deletion was only detected by read count CNV analysis using the tool ExomeDepth [26]. Thus, it is clear that there is added benefit in the multifaceted approach we applied so that important genetic diagnoses are not missed.

The association of the classical allele HLA-B*51 with BD is well-established and is considered the strongest genetic risk factor for BD [47]. Several other HLA genetic susceptibility loci for BD have also been identified [3]. Currently, in the UK, HLA genotyping is requested as a stand-alone test. Herein, we propose a combined genetic analysis to include WES and HLA genotyping, as a timesaving single test for all BD patients. We note that although OptiType was selected for our study [29], other HLA genotyping algorithms are available and could also be considered. Using this approach, we observed that 36% of the non-monogenic cases were HLA-B*51+, significantly higher than the allele frequency in the general population and providing further reassurance regarding the diagnosis of BD in this UK cohort.

Recent findings from monogenic diseases sharing features with BD such as HA20 strongly suggest a major role for dysregulated innate immunity activation due to variants in the NF-ĸB pathway in familial BD patients, while GWAS has also suggested rare polymorphisms in the NF-ĸB pathway contribute to genetic susceptibility in polygenic BD. HA20, due to novel heterozygous *TNFAIP3* variants (p.T76I, p. S548Dfs*128, p. M112Tfs*8, p. C657Vfs*14, p. E661Nfs*36), was the likely diagnosis in five cases included in this cohort. We highlight marked heterogenicity in the clinical presentation of these patients, with extremely variable treatment requirements and therapeutic responses ranging from glucocorticoids alone, to the need for systemic treatment involving different sequential biologic therapies (Table 1). This being the predominant monogenic mimic identified in this cohort, we emphasize the need to screen the *TNFAIP3* gene as a priority in the genetic work-up of BD.

Case 7 was found to possess a pathogenic variant in *TNFRSF13B* (p.A181E), which is associated with CVID. This diagnosis was supported by the patient having low IgA and IgM levels. Interestingly, the patient’s mother, who had a diagnosis of Sjögren’s syndrome, also harboured this variant. There are strong shared pathologies between Sjögren’s syndrome and CVID [48] and we therefore postulate that both mother and child have variable presentations of CVID associated with this pathogenic *TNFRSF13B* variant.

Case 15 was found to have ISG15 deficiency, an interferonopathy with a complex cellular and physiological phenotype caused by the interplay of ISG15 and both type I and II interferon signalling pathways. Patients typically experience susceptibility to mycobacterial disease, cerebral calcification, necrotizing skin lesions and other associated symptoms. This case had compound heterozygous deleterious variants, one novel nonsense variant (p.Q16X) and one multigene deletion encompassing *ISG15*. The latter was not detected using our germline variant caller but was identified using the read count analysis tool ExomeDepth [26]. This again highlights the importance of such multi-modal analyses when conducting monogenetic disease next-generation sequencing (NGS) analysis, as traditional variant callers do not have the capability to detect CNVs much larger than the NGS read length. Currently, this case is on a treatment-free trial period after her lesions resolved. Importantly, through genetic testing of the family members of this case, we identified both pathogenic variants in the infant younger sister of case 15, who at the time of writing was yet to develop symptoms. She is therefore being monitored regularly for any emergent features of ISG15 deficiency.

Of the seven novel proposed disease-causing variants identified across six families, six of these variants can be classified as pathogenic according to the ACMG criteria for classifying pathogenic variants [43]. For the remaining novel variant identified in case 5 (*TNFAIP3*: p.T76I) there is insufficient evidence to classify this variant as pathogenic, and as such this remains a variant of uncertain significance (VUS). In the context of this patient’s clinical and cellular phenotype (Supplementary Fig. S1, available at *Rheumatology* online), and recent reports of variably penetrant pathogenic missense *TNFAIP3* variants [49, 50] we believe this variant is likely influential in the patient’s disease, and as such have classified him as a monogenic case.

For the 22 non-monogenic cases, treatment consisted of the standard treatments for BD: glucocorticoids, azathioprine, colchicine, methotrexate, and anti-TNFα therapy all at standard doses. For these cases, a negative genetic result was important as it excluded a monogenic disease that may require a different therapeutic approach. It may be the case that in the future, genetic testing for BD can be provided at a national level, and we would suggest that analysis will require a multidisciplinary team approach of both skilled scientists and clinicians. The workflow provided herein provides a template that can be adopted accordingly for these purposes. In addition, centralized national testing would allow the creation of a large BD genetic sequencing dataset to facilitate continued understanding of BD genetics in the UK, and better therapeutic stratification.

Our study has several limitations. Firstly, 29% of patients in the cohort had monogenetic disease, somewhat higher than we had anticipated, possibly due to referral bias. Our study design attempted to minimize this by recruiting cases sequentially as they were referred, regardless of clinical presentation, or age of disease. We note, however, that most of the patients recruited were in fact cases with early-onset disease, which may have biased the results. Secondly, we only recruited patients from a UK cohort, so our findings may not be generalizable to other populations with a higher pre-test probability of typical non-monogenic BD. Additionally, filtering for disease-causing variants using trio or family WES data, although ideal, was often not possible for practical reasons in this multicentre study: frequently no parental DNA was available for adult patients; and the multicentre nature of the study meant tracking relatives from around the country was not feasible. Shortlisted variants were therefore only checked for familial segregation using Sanger sequencing or NGS where possible. We emphasize that in routine care, this is how genetic testing is usually performed in the UK, and elsewhere where resources are limited. We believe that this approach therefore makes our pipeline applicable in routine clinical care, where usually DNA from only the proband is available. Lastly, the genetic workflow applied in this study was not sensitive enough to detect large structural or pathogenic intronic variants that may cause monogenic disease, a recognized limitation of short-read WES in general.

In conclusion, we have described a combined genetic analysis approach for identifying monogenic mimics of BD and delivering HLA genotype for BD. A minority of this UK cohort had monogenic disease mimicking BD; the remainder could be classified as typical non-monogenic BD with a high proportion harbouring HLA-B*51. Our study now provides a strong rationale for such a combined genetic testing approach to be applied routinely in the clinical care of patients with BD.

## Supplementary Material

kead628_Supplementary_Data

## Data Availability

The data underlying this article are available on request.
