# Supplementary material for: Genetic testing of Behçet’s disease using next-generation sequencing to identify monogenic mimics and HLA-B*51
Source: Rheumatology (Oxford). 2023 Nov 25;63(12):3457–70. doi: 10.1093/rheumatology/kead628 (PMC11636563; doi:10.1093/rheumatology/kead628)
Supplement: kead628_Supplementary_Data [file kead628_supplementary_data.pdf]

Supplementary Table S1- Full description of International Criteria for Behçet's Disease (ICBD) [1], International Study Group (ISG) [2] and Paediatric Criteria for Behçet's Disease (PEDBD) [3].

**ICBD-** A score of 3 or more indicates a diagnosis of BD.	
Sign/symptom	***Points***
Ocular lesions	2
Oral aphthosis	2
Genital aphthosis	2
Skin lesions	1
Neurological manifestations	1
Vascular manifestations	1
Positive pathergy test*	1
**ISG-** These criteria are valid in absence of other clinical explanation.	
Required:	
Recurrent oral ulceration- minor aphthous, major aphthous, or herpetiform ulcers observed by the physician or patient, which have recurred at least 3 times over a 12-month period.	
Plus, any two of the following:	
Recurrent genital ulceration- Aphthous ulceration or scarring observed by the physician or patient	
Eye lesions- anterior uveitis, posterior uveitis, or cells in the vitreous on silt lamp examination, or retinal vasculitis detected by an ophthalmologist	
Skin lesions- erythema nodosum observed by physician or patient, pseudofolliculitis, or papulopustular lesions, or acneform nodules observed by the physician in a post adolescent patient who is not receiving corticosteroids	
Positive pathergy- test interpreted as positive by the physician at 24-48h	
**PEDBD-** Three of six items are required to classify a patient as having paediatric BD.	
Recurrent oral aphthosis- at least 3 attacks/year	
Genital ulceration- typically with scar	
Skin involvement- necrotic folliculitis, acneform lesions, erythema nodosum	
Ocular involvement- anterior or posterior uveitis, retinal vasculitis	
Neurological signs- with the exception of isolated headaches	
Vascular signs- venous thrombosis, arterial thrombosis, arterial aneurysm	

Supplementary Table S2- Inflammation panel gene list.

AARS2	*C4B*	*CORO1A*	*FBF1*	*IGHG2*	*LRRC8A*	*NLRP1*	*PRKG1*	*SH2D1A*	*TGFBR1*
ABCC6	*C5*	*COX10*	*FBLIM1*	*IGHM*	*LSM11*	*NLRP12*	*PRRT2*	*SH3BP2*	*TGFBR2*
ABI3	*C6*	*COX15*	*FBLN5*	*IGKC*	*LTBP2*	*NLRP3*	*PRX*	*SH3KBP1*	*THBD*
ACD	*C7*	*CPT2*	*FBN1*	*IGLL1*	*LYN*	*NLRP6*	*PSAP*	*SKI*	*TICAM1*
ACOX1	*C8A*	*CR2*	*FBN2*	*IKBKB*	*LYST*	*NLRP7*	*PSEN1*	*SKIV2L*	*TINF2*
ACP5	*C8B*	*CSF1R*	*FBRS*	*IKBKG*	*LYZ*	*NOD2*	*PSENEN*	*SLC13A3*	*TIRAP*
ACTA2	*C8G*	*CSF2*	*FCGR1A*	*IKZF1*	*MAGT1*	*NOP10*	*PSMA3*	*SLC13A4*	*TLR3*
ACTB	*C9*	*CSF2RA*	*FCGR2A*	*IKZF3*	*MALT1*	*NOS2*	*PSMB10*	*SLC16A2*	*TLR7*
ACVRL1	*CARD10*	*CSF2RB*	*FCGR2B*	*IL10*	*MAP1LC3B2*	*NOTCH1*	*PSMB3*	*SLC17A5*	*TLR8*
ADA	*CARD11*	*CSF3R*	*FCGR3A*	*IL10RA*	*MAP3K14*	*NOTCH3*	*PSMB4*	*SLC1A4*	*TMC6*
ADA2	*CARD14*	*CST3*	*FCGR3B*	*IL10RB*	*MAPK8*	*NPC1*	*PSMB8*	*SLC25A12*	*TMC8*
ADAM17	*CARD9*	*CTC1*	*FCGRT*	*IL12B*	*MASP1*	*NRAS*	*PSMB9*	*SLC29A3*	*TMEM107*
ADAMTS10	*CARMIL2*	*CTLA4*	*FCHO1*	*IL12RB1*	*MASP2*	*NSMCE3*	*PSMG2*	*SLC2A10*	*TMEM173*
ADAMTS17	*CASP10*	*CTNNBL1*	*FCN3*	*IL12RB2*	*MAT2A*	*NUBPL*	*PSTPIP1*	*SLC35C1*	*TNFAIP3*
ADAMTS2	*CASP8*	*CTPS1*	*FERMT1*	*IL17A*	*MBL2*	*OAS1*	*PTEN*	*SLC37A4*	*TNFRSF11A*
ADAMTSL2	*CBL*	*CTSC*	*FERMT3*	*IL17F*	*MCM10*	*OCLN*	*PTPN2*	*SLC39A13*	*TNFRSF13B*
ADAMTSL4	*CBS*	*CXCR2*	*FGA*	*IL17RA*	*MCM4*	*ODC1*	*PTPN22*	*SLC39A7*	*TNFRSF13C*
ADAR	*CCBE1*	*CXCR4*	*FKBP14*	*IL17RC*	*MED13L*	*ORAI1*	*PTPRC*	*SLC46A1*	*TNFRSF1A*
AGR2	*CD19*	*CXorf36*	*FLNA*	*IL18*	*MEFV*	*OSMR*	*PYCARD*	*SLC7A7*	*TNFRSF4*
AICDA	*CD247*	*CYBA*	*FNIP1*	*IL18BP*	*MFAP5*	*OSTM1*	*PYCR2*	*SLC9A3*	*TNFRSF9*
AIFM1	*CD27*	*CYBB*	*FOLR1*	*IL1RN*	*MFN2*	*OTULIN*	*RAB27A*	*SMAD2*	*TNFSF11*
AIMP1	*CD28*	*CYP27A1*	*FOXE3*	*IL21*	*MICA*	*PARN*	*RAC2*	*SMAD3*	*TNFSF12*
AIRE	*CD3D*	*DARS*	*FOXM1*	*IL21R*	*MKL1*	*PARP1*	*RAD50*	*SMAD4*	*TNFSF13*
AK2	*CD3E*	*DARS2*	*FOXN1*	*IL22*	*MLC1*	*PAX1*	*RAG1*	*SMAD6*	*TNIP1*
ALDH3A2	*CD3G*	*DBR1*	*FOXP3*	*IL23A*	*MLPH*	*PDCD1*	*RAG2*	*SMARCAL1*	*TNXB*
ALPI	*CD4*	*DCLRE1B*	*FPR1*	*IL23R*	*MOGS*	*PDHA1*	*RANBP2*	*SMARCD2*	*TOM1*
ALPK1	*CD40*	*DCLRE1C*	*FPR2*	*IL2RA*	*MPEG1*	*PEPD*	*RARS*	*SNORA31*	*TOP2B*
AP1S3	*CD40LG*	*DDX58*	*FPR3*	*IL2RB*	*MPI*	*PEX1*	*RASGRP1*	*SNORD118*	*TPK1*
AP3B1	*CD46*	*DEF6*	*FUCA1*	*IL2RG*	*MPLKIP*	*PEX10*	*RBCK1*	*SNX10*	*TPP1*
AP3D1	*CD55*	*DGUOK*	*FXN*	*IL31RA*	*MPO*	*PEX11A*	*RC3H1*	*SOCS1*	*TPP2*
APOA1	*CD59*	*DKC1*	*G6PC3*	*IL36RN*	*MPZ*	*PEX11B*	*RECQL4*	*SORT1*	*TRAC*
APOA2	*CD70*	*DKCA1*	*G6PD*	*IL37*	*MR1*	*PEX12*	*REL*	*SOX10*	*TRAF3*
APOA4	*CD79A*	*DNAJC21*	*GAD1*	*IL6*	*MRE11*	*PEX13*	*RELA*	*SP110*	*TRAF3IP2*
APOC2	*CD79B*	*DNASE1*	*GALC*	*IL6R*	*MRPS16*	*PEX14*	*RELB*	*SPI1*	*TRAP1*
APOC3	*CD81*	*DNASE1L3*	*GATA1*	*IL6ST*	*MS4A1*	*PEX16*	*RELN*	*SPINK5*	*TREX1*
APOE	*CD8A*	*DNASE2*	*GATA2*	*IL7R*	*MSH6*	*PEX2*	*RET*	*SPPL2A*	*TRIM22*
APOL1	*CDC42*	*DNMT1*	*GBE1*	*INO80*	*MSN*	*PEX26*	*RFX5*	*SPTAN1*	*TRIM28*
APP	*CDCA7*	*DNMT3B*	*GFAP*	*IPO8*	*MTHFD1*	*PEX3*	*RFXANK*	*SRP54*	*TRNT1*
ARHGAP42	*CEBPE*	*DOCK2*	*GFI1*	*IRAK1*	*MTPAP*	*PEX5*	*RFXAP*	*STAT1*	*TSC1*
ARHGEF1	*CFB*	*DOCK8*	*GFM1*	*IRAK4*	*MVK*	*PEX6*	*RGS10*	*STAT2*	*TSC2*
ARPC1B	*CFD*	*DYSF*	*GIMAP5*	*IRF2BP2*	*MYD88*	*PEX7*	*RHOD*	*STAT3*	*TSPAN14*
ARSA	*CFH*	*EARS2*	*GIMAP6*	*IRF3*	*MYH11*	*PGM3*	*RHOG*	*STAT4*	*TTC37*
ARX	*CFHR1*	*ECM1*	*GINS1*	*IRF4*	*MYLK*	*PHYH*	*RHOH*	*STAT5A*	*TTC7A*
ASPA	*CFHR2*	*EFEMP2*	*GJB1*	*IRF7*	*MYO5A*	*PI4KA*	*RIN2*	*STAT5B*	*TTR*
ATG4A	*CFHR3*	*EFL1*	*GJC2*	*IRF8*	*MYO5B*	*PIK3CD*	*RIPK1*	*STIM1*	*TUBB4A*
ATM	*CFHR4*	*EGR2*	*GLA*	*IRF9*	*MYOF*	*PIK3CG*	*RMRP*	*STK4*	*TUBGCP3*
ATP6AP1	*CFHR5*	*EIF2B1*	*GSN*	*ISCA2*	*MYSM1*	*PIK3R1*	*RNASEH2A*	*STN1*	*TUFM*
ATP6V0A2	*CFI*	*EIF2B2*	*GTF2H5*	*ISG15*	*NBAS*	*PLCG2*	*RNASEH2B*	*STX11*	*TYK2*
ATP7B	*CFP*	*EIF2B3*	*GUCY1A1*	*ITCH*	*NBN*	*PLEKHM1*	*RNASEH2C*	*STX1B*	*TYMP*
ATPAF2	*CFTR*	*EIF2B4*	*GUCY1A3*	*ITGAM*	*NCF1*	*PLG*	*RNASET2*	*STXBP1*	*UBA1*
B2M	*CHD7*	*EIF2B5*	*GUCY2C*	*ITGB2*	*NCF2*	*PLOD1*	*RNF168*	*STXBP2*	*UNC119*
B3GALT6	*CHRNB2*	*ELANE*	*HAVCR2*	*ITK*	*NCF4*	*PLOD3*	*RNF213*	*STXBP3*	*UNC13D*
B4GALT7	*CHST14*	*ELF4*	*HAX1*	*ITPKB*	*NCKAP1L*	*PLP1*	*RNF31*	*SUCLA2*	*UNC93B1*

BACH2	*CIB1*	*ELN*	*HELLS*	*IVNS1ABP*	*NCSTN*	*PMP22*	*RNU4ATAC*	*SUMF1*	*UNG*
BCL10	*CIITA*	*EPCAM*	*HEPACAM*	*JAGN1*	*NDUFAF1*	*PMS2*	*RNU7-1*	*SURF1*	*USB1*
BCL11B	*CLCN2*	*EPG5*	*HFE*	*JAK1*	*NDUFS1*	*PNP*	*RORC*	*SYK*	*USP18*
BCS1L	*CLCN7*	*ERBIN*	*HMBS*	*JAK3*	*NDUFS2*	*POLA1*	*RPSA*	*TACO1*	*VPS13B*
BGN	*CLEC16A*	*ERCC2*	*HMOX1*	*KDM6A*	*NDUFS3*	*POLD1*	*RRM2B*	*TAP1*	*VPS45*
BLM	*CLPB*	*ERCC3*	*HPS1*	*KMT2A*	*NDUFS4*	*POLD2*	*RTEL1*	*TAP2*	*WAS*
BLNK	*CNBP*	*ERCC4*	*HPS4*	*KMT2D*	*NDUFS5*	*POLE*	*SAMD3*	*TAPBP*	*WDR1*
BLOC1S6	*CNTN3*	*ERCC6*	*HPS6*	*KRAS*	*NDUFS6*	*POLE2*	*SAMD9*	*TAZ*	*WIPF1*
BMPR2	*COL11A1*	*ERCC6L2*	*HS3ST6*	*LACC1*	*NDUFS7*	*POLG*	*SAMD9L*	*TBC1D24*	*WRAP53*
BOLA3	*COL1A1*	*ERCC8*	*HSD17B4*	*LAMTOR2*	*NDUFS8*	*POLG2*	*SAMHD1*	*TBK1*	*XIAP*
BRCA1	*COL1A2*	*ETFDH*	*HTR1A*	*LAT*	*NDUFV1*	*POLR1C*	*SART3*	*TBX1*	*YY1AP1*
BRCA2	*COL2A1*	*EXOSC8*	*HTRA1*	*LCK*	*NEFL*	*POLR3A*	*SASH3*	*TBX21*	*ZAP70*
BTD	*COL3A1*	*EXTL3*	*HTRA2*	*LCP2*	*NF1*	*POLR3B*	*SBDS*	*TCF3*	*ZBTB24*
BTK	*COL4A1*	*F12*	*HYOU1*	*LIG1*	*NFAT5*	*POLR3C*	*SCN9A*	*TCIRG1*	*ZC3HC1*
C17orf62	*COL4A5*	*FAAP24*	*IBA57*	*LIG4*	*NFE2L2*	*POLR3F*	*SCO1*	*TCN2*	*ZFP36*
C1QA	*COL5A1*	*FADD*	*ICOS*	*LMNA*	*NFKB1*	*POMP*	*SCO2*	*TERC*	*ZNF34*
C1QB	*COL5A2*	*FAM126A*	*ICOSLG*	*LMNB1*	*NFKB2*	*POU2AF1*	*SCP2*	*TERT*	*ZNF341*
C1QC	*COL7A1*	*FANCF*	*IFIH1*	*LONP1*	*NFKBIA*	*PRDM5*	*SDHAF1*	*TET2*	*ZNF469*
C1R	*COLEC11*	*FANCI*	*IFNAR1*	*LOX*	*NFKBID*	*PRF1*	*SDHB*	*TFRC*	*ZNFX1*
C1S	*COPA*	*FANCM*	*IFNAR2*	*LPIN2*	*NFU1*	*PRG4*	*SEC61A1*	*TGFB1*	
C2	*COQ2*	*FAS*	*IFNG*	*LRBA*	*NHEJ1*	*PRIM1*	*SEMA3E*	*TGFB2*	
C3	*COQ8A*	*FASLG*	*IFNGR1*	*LRP1*	*NHP2*	*PRKCD*	*SERPING1*	*TGFB3*	
C4A	*COQ9*	*FAT4*	*IFNGR2*	*LRRC32*	*NLRC4*	*PRKDC*	*SGPL1*	*TGFBI*	

Supplementary Table S3- Comparison of patient scores for the International Criteria for Behçet's Disease (ICBD) [1], International Study Group (ISG) [2] and Paediatric Criteria for Behçet's Disease (PEDBD) [3]. PEDBD scores were not determined for adult patients. Y indicates the patient met the criteria; N indicates they did not.

Case	ICBD Y/N (Score)	ISG Y/N	PEDBD Y/N (Score)
1	Y (5)	N	Y (3)
2	Y (6)	Y	Y (3)
3	Y (5)	N	N (2)
4	Y (6)	Y	N/A
5	Y (4)	N	N/A
6	N (3)	N	N (1)
7	Y (7)	Y	Y (4)
8	Y (5)	N	Y (3)
9	Y (4)	N	N (2)
10	Y (4)	N	N (2)
11	Y (5)	Y	Y (3)
12	Y (6)	Y	N/A
13	Y (4)	N	N (2)
14	Y (4)	N	N (2)
15	N (3)	N	N (2)
16	Y (6)	Y	Y (4)
17	Y (6)	Y	Y (4)
18	Y (4)	N	N (2)
19	Y (7)	Y	N/A
20	Y (7)	Y	Y (3)
21	Y (5)	Y	Y (3)
22	Y (5)	Y	Y (4)
23	Y (4)	N	N (2)
24	Y (4)	N	N (2)
25	Y (6)	N	N/A
26	Y (5)	Y	Y (3)
27	Y (7)	Y	N/A
28	Y (7)	N	N/A
29	Y (4)	N	N (2)
30	Y (5)	Y	N/A
31	N (3)	N	N (2)

Supplementary Table S4- Complete HLA type I genotypes of all cases.

**Case**	**HLA-A**		**HLA-B**		**HLA-C**	
1	A*24:02	A*03:01	B*07:05	B*49:01	C*15:05	C*07:01
2	A*24:03	A*01:01	B*18:01	B*15:17	C*12:03	C*07:01
3	A*01:01	A*29:02	**B*51:01**	B*57:01	C*03:03	C*06:02
4	A*02:11	A*02:11	B*35:03	B*40:06	C*15:02	C*04:01
5	A*01:01	A*03:01	B*07:02	B*35:02	C*07:02	C*04:01
6	A*03:01	A*02:05	B*50:01	B*18:01	C*12:03	C*06:02
7	A*24:02	A*01:01	B*08:01	B*15:01	C*03:03	C*07:01
8	A*02:01	A*02:01	B*44:02	B*15:01	C*03:03	C*05:01
9	A*02:01	A*01:01	B*08:01	**B*51:01**	C*07:01	C*05:01
10	A*02:01	A*29:02	B*44:05	B*44:03	C*16:01	C*02:02
11	A*01:01	A*03:01	B*08:01	B*15:01	C*03:03	C*07:01
12	A*02:01	A*68:01	**B*51:01**	B*15:01	C*03:04	C*14:02
13	A*02:01	A*11:01	B*35:01	B*40:01	C*03:04	C*04:01
14	A*02:11	A*24:02	B*40:06	B*35:03	C*12:03	C*15:02
15	A*26:01	A*01:01	B*44:03	B*40:01	C*03:04	C*04:01
16	A*01:03	A*68:02	B*07:02	B*07:02	C*07:02	C*07:02
17	A*01:03	A*03:01	B*07:02	B*14:02	C*07:02	C*08:02
18	A*24:02	A*02:17	B*07:02	**B*51:01**	C*07:02	C*15:02
19	A*01:01	A*68:01	B*08:01	**B*51:01**	C*16:02	C*07:01
20	A*31:01	A*68:01	B*15:17	B*40:01	C*03:04	C*07:01
21	A*24:02	A*02:01	B*07:02	B*40:01	C*03:04	C*04:01
22	A*24:02	A*02:01	B*07:02	**B*51:01**	C*14:02	C*04:01
23	A*24:02	A*26:01	**B*51:01**	B*38:01	C*12:03	C*15:06
24	A*24:02	A*11:01	B*35:01	B*49:01	C*07:01	C*04:01
25	A*02:01	A*32:01	B*15:03	B*40:01	C*03:04	C*02:10
26	A*33:03	A*02:11	B*15:05	B*44:03	C*03:03	C*07:01
27	A*30:02	A*02:11	B*15:05	B*53:01	C*03:03	C*04:01
28	A*31:01	A*24:02	**B*51:01**	B*15:01	C*03:03	C*16:02
29	A*01:01	A*03:01	B*08:01	B*15:01	C*01:02	C*07:01
30	A*31:01	A*02:01	B*15:01	B*35:01	C*03:03	C*04:01
31	A*02:01	A*01:01	B*55:01	B*57:01	C*03:03	C*06:02

Supplementary Figure S1- NF-κB signalling assay to investigate pathogenicity of novel *TNFAIP3* mutations in cases 5, 14, 29 and 31. Expression of phosphorylated p65, a component of the NF-κB transcription factor complex which becomes phosphorylated upon pathway activation, was quantified by flow cytometry in patient and healthy control lymphocytes after time course stimulation with TNFα. For healthy control samples (n=3) data are displayed as mean ±SEM. Cases 5, 14, and 31 showed increased pathway activation upon stimulation. Case 29 showed less activation; however, this was likely due to his treatment with anti-TNFα and ustekinumab. His mother, who also harboured the mutation but was untreated, showed increased activation as for the other cases. Abbreviations; MFI, median fluorescence intensity; WT, wildtype; *TNFAIP3*, Tumour necrosis factor alpha-induced protein 3; TNFα, Tumour necrosis factor alpha; min, minutes.

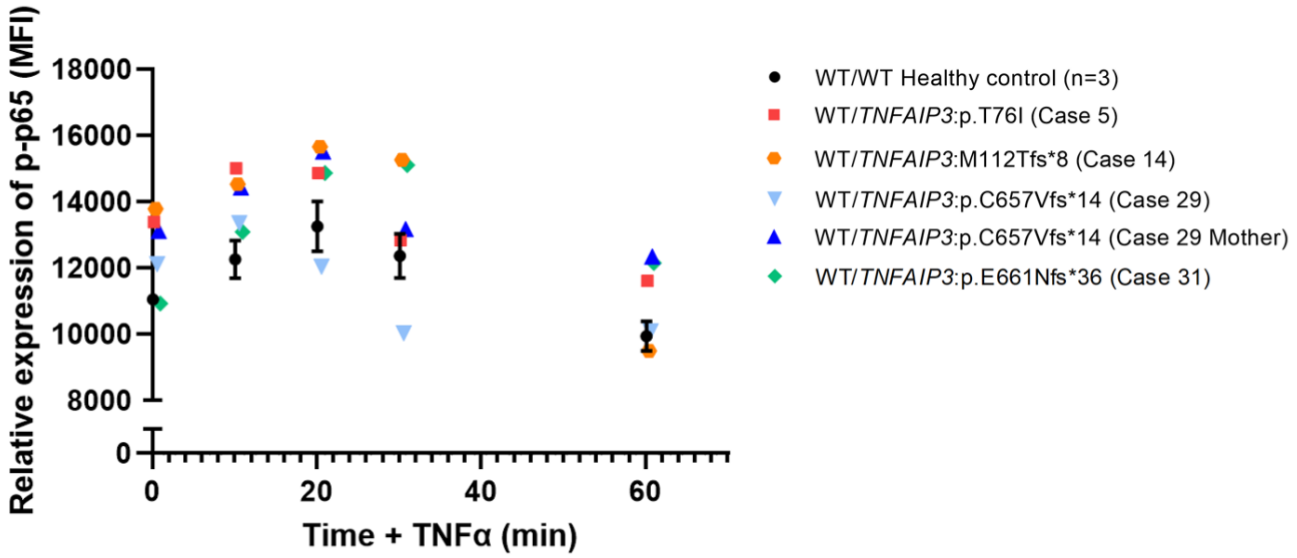

Supplementary Figure S2- Pedigrees of families with suspected monogenic disease. Probands are indicated by an arrow.

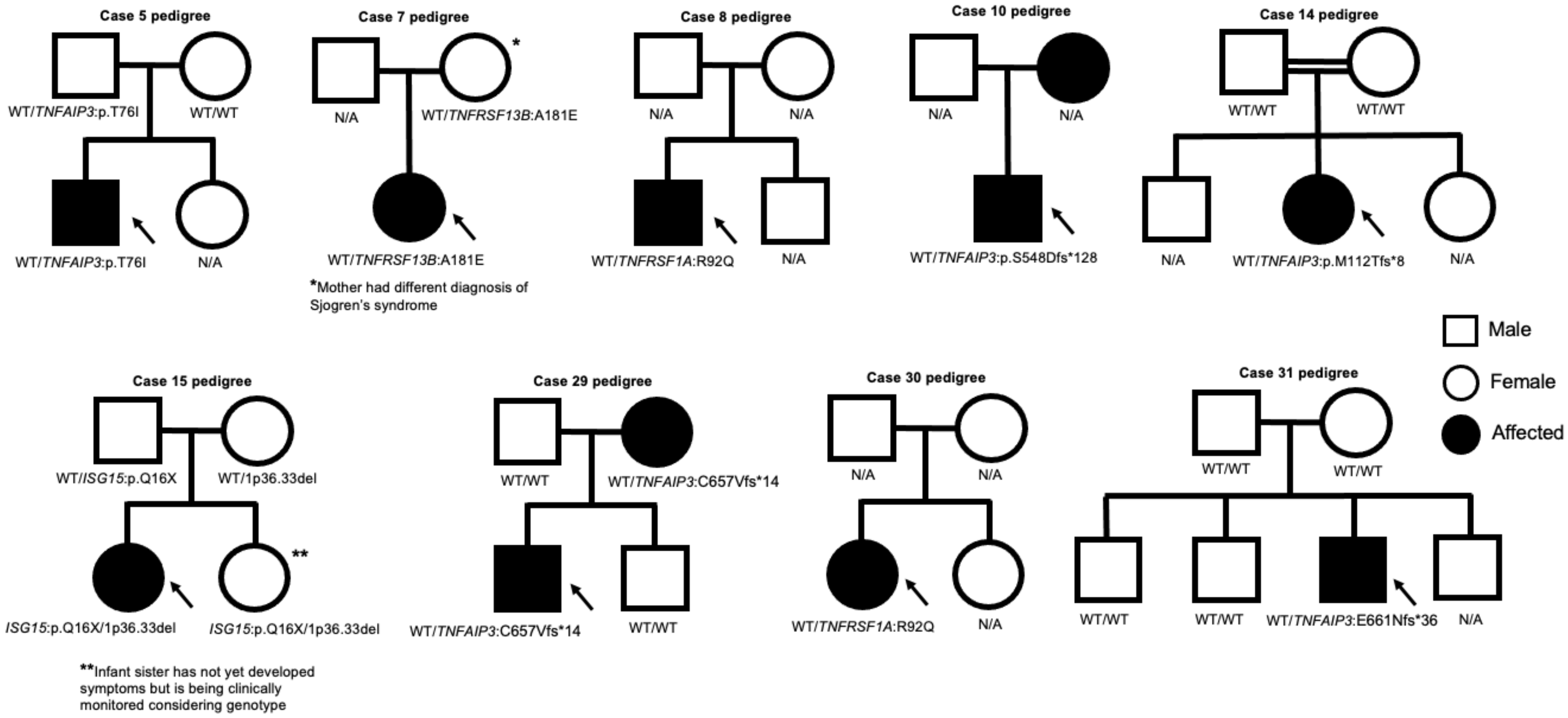

References

1. Davatchi, F., et al., *The International Criteria for Behçet's Disease (ICBD): a collaborative study of 27 countries on the sensitivity and specificity of the new criteria.* Journal of the European Academy of Dermatology and Venereology, 2014. **28**(3): p. 338-347.
2. International Study Group for Behçet's, D., *Criteria for diagnosis of Behcet's disease.* The Lancet, 1990. **335**(8697): p. 1078-1080.
3. Koné-Paut, I., et al., *Consensus classification criteria for paediatric Behçet's disease from a prospective observational cohort: PEDBD.* Annals of the Rheumatic Diseases, 2016. **75**(6): p. 958-964.
